# Life after paediatric intensive care unit

**DOI:** 10.7196/AJTCCM.2019.v25i4.027

**Published:** 2019-12-06

**Authors:** S T Hlophe, R Masekela

**Affiliations:** Department of Paediatrics and Child Health, Nelson R Mandela School of Clinical Medicine, University of KwaZulu-Natal, Durban, South Africa

**Keywords:** post, PICU syndrome, life after

## Abstract

Advances in critical care medicine have led to reduced mortality but increased morbidity. Post-intensive care unit syndrome (PICS) develops after critical illness and presents as cognitive, physical and/or psychosocial impairments. PICS is prevalent in 10 - 36% of patients after discharge from paediatric intensive care unit. Multiple risk factors are associated with PICS, but there is no single causal factor. Factors range from clinical illnesses to intensive care intervention. The care plan should be aimed at prevention, early identification and post-ICU management of PICS by a multidisciplinary team.

## Background

Post-intensive care unit syndrome (PICS) is a condition that develops after critical illness that presents as impaired cognitive, physical or mental health status that may persist beyond hospitalisation.^[1]^ Advances in critical care medicine and the evolution of life-saving interventions has led to reduced mortality. While more seriously ill patients are saved, a significant increase has been observed in functional disabilities, secondary to critical illness and management.^[2]^ Success in intensive care unit (ICU) management should not be limited to patient survival to discharge but should take into account the post-ICU functional status. Critical illness, especially in cases of multi-organ dysfunction, is associated with muscle weakness which ffects both short- and long-term outcomes.^[3]^ The impact of critical illnesses extends beyond patients, affecting families and healthcare providers.

A multi-disciplinary management plan to improve the long-term functioning capacity and quality of life of ICU survivors and their families is required.^[2]^ A physical manifestation of PICS is muscle weakness, which is reported to be associated with steroid use and mechanical ventilation.^[4–7]^ In addition to muscle weakness, neuromuscular complications are common and represent a major functional defect.^[8]^ Furthermore, psychological and emotional dysfunction can persist for up to 5 years in both patients and families affected.^[9]^ Therefore, it is critical that post-ICU follow-ups are routinely done to review and assess for PICS-related complications or the management received during ICU stay. It is not always possible to assess the premorbid status of a patient prior to the paediatric intensive care unit (PICU), therefore the patient’s dysfunction should be assessed based on the history from the caregiver or by comparisons with patient groups of similar age.

## Epidemiology

The acquired functional impairment ranges between 10 and 36% of patients at discharge and 10 and 13% 2 years after discharge.^[4-8,10]^

## Risk factors

While there is no single causal factor, some risk factors have been reported to be associated with PICS (Table 1). Patients at high risk are those with prolonged mechanical ventilation (>7 days), sepsis, multisystem organ failure and prolonged duration of deep sedation. ^[2,6,7]^ Other risk factors include prior cognitive impairment, severe critical illness and pre-existing disabilities.^[11]^ A systematic review of 18 studies found that the use of corticosteroids was significantly associated with increased odds of developing ICUassociated weakness.^[4]^ The weakness is frequent during recovery from critical illness and it may be persistent amongst PICU survivors.^[10]^

**Table 1:** Risk factors for PICS^[2,6,7,11,14]^

Group	Risk factors
Clinical	Severe critical illness
	Sepsis
	Multi-organ dysfunction
	Immobility
	Acute respiratory distress syndrome
	Glucose dysregulation
	Acute brain dysfunction
ICU interventions	Benzodiazepines
	Opioids
	Muscle relaxants
	Early parenteral nutrition
	Glucocorticoids
	Prolonged mechanical ventilation
	Renal replacement therapy use
Other	ICU length of stay >7 days

PICS = post-intensive care unit syndrome

ICU = intensive care unit

## Types of impairment

The types of impairments observed in those that survive critical illness can be categorised in 3 broad categories (summarised in Table 2).

**Table 2:** Clinical presentation ^[2,7,9,11-17]^

Impairment	Presentation
Physical	Poor mobility
	Recurrent falls
	Muscle wasting
	Weight loss
	Impaired lung function
	Poor feeding
	Early extubation
Cognitive	Behavioural disorder
	Memory loss
	Poor school performance
Psychosocial	Post-traumatic stress disorder
	Sleep disturbance
	Mood disorder
	Parental depression/stress/anxiety
	Family dysfunction/grief

### Physical impairment

The physical impairments observed among a proportion of ICU survivors include poor mobility, recurrent falls and tetraplegia.^[2,12]^ Muscle wasting is also seen in critically ill patients and is secondary to multiple causes such as inadequate nutrition, neuropathy, myopathy, sepsis and drug therapy.^[11]^ Neuromuscular weakness, weight loss and impaired lung function are other physical manifestations seen in those who survive critical illness.^[11]^ After intensive care, one in five children are in need of some form of specialist care such as speech therapy, private schooling or physiotherapy for physical rehabilitation.^[11,13]^ Functional impairment is often a barrier to carrying out routine daily activities, affecting feeding, mobility and self-care. Neurological examinations, electrophysiological measurements, serum creatinine kinase levels and muscle biopsies are useful tools in the identification and characterisation of the polyneuropathy and myopathy.^[7]^

### Cognitive impairment

 Cognitive impairment presents as difficulty performing tasks at home or at school due to behavioural or neuropsychological difficulties. When compared to healthy children, post-ICU children had impaired memory, particularly those admitted with traumatic brain injury, meningoencephalitis or sepsis.^[14]^

### Psychosocial impairment

School-going children and adolescents are at risk for post-traumatic stress disorder after critical illness.^[15]^ Other presentations include sleep disturbances and mood disorder.^[1,15]^ Illness severity in children has detrimental effects on maternal stress levels, often increasing with the duration of PICU stay.^[16]^ In addition, stress levels in the mother of an ICU child correlate with the distance of the ICU unit from the family home, duration of mechanical ventilation and the absence of psychosocial support services offered while in ICU.^[16]^ These stresses extend to the entire family, requiring resiliency and adaptation by family members which places a huge burden on normal family functioning.^[16]^ Family members and/or caregivers experience psychological dysfunction and complicated griefwhen their loved ones are admitted to PICU or discharged with complications.^[17]^ This in turn may have negative effects on patients’ ability to recovery and rehabilitate if not identified and treated early.^[9]^ Post-ICU management should be family-centred and cater to the specific needs of each individual.

## Prevention and management

A multi-disciplinary approach, harnessing the expertise of physiotherapists, occupational therapists, psychiatrists, neurologists and rehabilitation specialists to manage post-ICU survivors with the impairments.^[18–21]^ Concomitant psycho-social support should be available to caregivers during and after PICU admission. Post-ICU follow-ups with a multi-disciplinary approach to managing patient morbidities will allow more appropriate referrals to be made, ultimately benefiting those with multisystem dysfunction.^[22]^ This way, the quality and long-term impact of ICU care can be evaluated.^[22]^ Prevention and management strategies of PICS can be grouped into three categories; physical, cognitive and psychosocial (summarised in Table 3).

**Table 3:** Prevention and management^[1,3,7,11,15,19,21,23-26]^

Impairment	Intensive care unit (ICU)	Post ICU
Physical	Early mobilisation	Early rehabilitation by a multidisciplinary team
	Glucose control	Adequate nutrition
	Awakening and breathing trial daily	Minimal sedation and muscle relaxants
	Minimal sedation/avoid deep sedation	
	Early screening for muscle weakness	
	Adequate nutrition	
	Low-dose, short-duration glucocorticosteroid use	
Cognitive	Diary	Social support (social grants)
	Glucose control	Special-need school
	Prevent hypoxia	
	Neuroprotection	
Psychosocial	Source control/infection control	Counselling and education
	Screening for post-traumatic symptoms	Psychiatric evaluation and management
	Minimal sedation	Parents’ involvement in decision-making
	Psychiatric evaluation and management	
	Counselling and education	
	Parents’ involvement in decision-making	

### Physical

The goal of preventing and managing PICS should be focused on minimising complications and stressors, secondary to the interventions delivered within the ICU.^[3,23]^ Early mobilisation is reported to significantly reduce the odds of weakness on hospital discharge by 82% (*p*=0.003).^[5]^ After PICU admission, active or passive exercises are recommended to facilitate early mobilisation and rehabilitation by physiotherapists, occupational therapists and palliative care team.^[11,19,21]^ The use of the ABCDE bundle (Awakening and Breathing Coordination, Delirium monitoring and management, Early mobilisation) has shown good preventive rates for PICS.^[3,23-25]^ Awakening is done by using light or minimal sedation. Both sedative and opioid over-use is associated with numerous adverse outcomes during and after critical illness.^[3,23-25]^ These adverse outcomes include an increase in the duration of mechanical ventilation, prolonged stay in the ICU and risk of nosocomial complications.^[7,24]^ Limiting the use of deep sedation and early mobilisation are important preventive strategies that demonstrate a positive impact in preventing the long-term functional disabilities associated with PICS.^[19,25]^ Early rehabilitation can prevent acute skeletal muscle mass loss experienced in some survivor children. ^[11,21]^ Screening for clinical muscle weakness in patients with prolonged ICU admission (>7 days) allows early identification of patients with ICU-acquired weakness and identifies those at risk for short-term morbidity and mortality in the long term (up to one year after the acute event).^[8]^ It has been reported that mobilising mechanically ventilated patients is both feasible and beneficial.^[24]^ Hyperglycaemia has been associated with poor outcomes, including increased infectious complications, physical weakness and mortality, and therefore it is vital to maintain euglycaemia during ICU stay.^[5]^

### Cognitive

Hypoxia and hypoglycaemia prevention and management should be prioritised, especially for the high-risk group (e.g. acute brain injury), because they are reported as major risk factors for neurocognitive impairment.^[11]^ Neuroprotection to prevent secondary brain injury especially for patients with traumatic brain injury, is advised. Younger children of low socioeconomic status are at risk for greater neurocognitive impairment at the initial post-ICU visit.^[1]^ Prolonged use of drugs (e.g. sedatives), particularly at high doses, should be avoided as they are associated with neurocognitive adverse events.^[3]^ In post-ICU survivor children, especially children who were admitted with meningoencephalitis or septic illness, additional support to carry out daily activities and tasks is required.^[26]^ A manual record of all the morbidities experienced in post-survivor children in the home is recommended.

### Psychosocial

Healthcare providers in PICU should, as a matter of priority, pay close attention to risk factors for neurocognitive impairment such as septic illness, invasive interventions and benzodiazepines.^[1]^ Psychological assessment and management for post-traumatic stress disorder (PTSD) after ICU discharge is vital. The PTSD symptoms most frequently reported by PICU patients include the inability to recall aspects of the event, hypervigilance, avoidance of thoughts or feelings, physiological reactivity to trauma reminders, and intrusive thoughts or images.^[15]^ Screening tools that determine the presence or absence of post-traumatic stress symptoms are important.^[1]^ ICU management needs to be family-centred: this includes respecting the needs and preferences of the individual families, counselling of caregivers, support groups and social worker and psychologist’s involvement during and after PICU admission. It is important to provide full information about the patient’s diagnosis and involve the family in the management planning. Involving parents in decision making and meeting their needs for emotional support is of utmost importance in the case of children with PICS.

## Conclusion

Preventing and mitigating the morbidities in ICU is just as important as reducing mortality. The goal of ICU management should be to discharge the patient in a condition similar to their pre-admission status. All measures should be put in place to prevent ICU-acquired complications. Among others, these include the limited use of muscle relaxants (of low dose and short duration), multidisciplinary team efforts to assess and intervene with early rehabilitation where needed and early mobilisation. Psychological support for the family and the patient from the time of admission is recommended. In addition, caregivers should also be evaluated during follow-up visits and referred for psychosocial support when necessary. A post-ICU follow-up at a clinic should be conducted to help identify and manage a patient with post-ICU complications which may have been missed on discharge. All PICUs should have a follow-up clinic that involves a multidisciplinary team previously involved in the care of the patient.
